# *catena*-Poly[[copper(II)-bis[μ-bis(3,5-dimethyl-1*H*-pyrazol-4-yl) selenide]] bis(perchlorate)]

**DOI:** 10.1107/S1600536809042056

**Published:** 2009-10-23

**Authors:** Maksym Seredyuk, Matti Haukka, Vadim A. Pavlenko, Igor O. Fritsky

**Affiliations:** a National Taras Shevchenko University, Department of Chemistry, Volodymyrska str. 64, 01033 Kyiv, Ukraine; b Department of Chemistry, University of Joensuu, PO Box 111, 80101 Joensuu, Finland

## Abstract

In the title compound, {[Cu(C_10_H_14_N_4_Se)_2_](ClO_4_)_2_}_*n*_, the Cu^II^ ion is located on a twofold rotation axis and has a tetra­gonally distorted square-planar geometry constituted by four N atoms. A pair of bis(3,5-dimethyl-1*H*-pyrazol-4-yl) selenide (*L*) ligands bridges the copper centers into a polymeric chain extending along [001]. The perchlorate anions are involved in inter­molecular N—H⋯O hydrogen bonding, which links the chains into layers parallel to the *bc* plane.

## Related literature

For the potential applications of coordination polymers, see: Farha *et al.* (2009 ▶); Ohba *et al.* (2009 ▶); Shibahara *et al.* (2007 ▶). For our studies of similar complexes with different dimensionality, see Seredyuk *et al.* (2007 ▶).
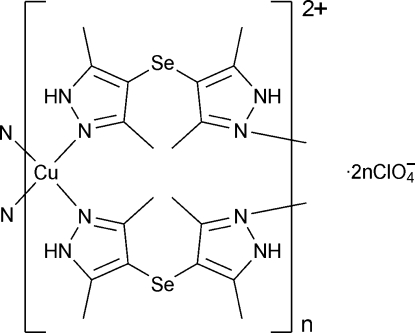


## Experimental

### 

#### Crystal data

[Cu(C_10_H_14_N_4_Se)_2_](ClO_4_)_2_
*M* *_r_* = 800.86
Monoclinic, 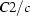

*a* = 28.398 (6) Å
*b* = 7.5865 (15) Å
*c* = 18.517 (4) Å
β = 130.69 (3)°
*V* = 3025.1 (17) Å^3^
*Z* = 4
Mo *K*α radiation
μ = 3.36 mm^−1^
*T* = 120 K
0.20 × 0.15 × 0.05 mm

#### Data collection

Nonius KappaCCD diffractometer
Absorption correction: multi-scan (*SADABS*; Sheldrick, 1996 ▶) *T*_min_ = 0.552, *T*_max_ = 0.845
13077 measured reflections
3415 independent reflections
2799 reflections with *I* > 2σ(*I*)
*R*_int_ = 0.074

#### Refinement

*R*[*F* ^2^ > 2σ(*F* ^2^)] = 0.047
*wR*(*F* ^2^) = 0.115
*S* = 1.04
3415 reflections
191 parameters
H-atom parameters constrained
Δρ_max_ = 2.18 e Å^−3^
Δρ_min_ = −1.00 e Å^−3^

### 

Data collection: *COLLECT* (Bruker–Nonius, 2004 ▶); cell refinement: *DENZO*/*SCALEPACK* (Otwinowski & Minor, 1997 ▶); data reduction: *DENZO*/*SCALEPACK*; program(s) used to solve structure: *SHELXS97* (Sheldrick, 2008 ▶); program(s) used to refine structure: *SHELXL97* (Sheldrick, 2008 ▶); molecular graphics: *ORTEP-3 for Windows* (Farrugia, 1997 ▶); software used to prepare material for publication: *DIAMOND* (Brandenburg, 2006 ▶).

## Supplementary Material

Crystal structure: contains datablocks I, global. DOI: 10.1107/S1600536809042056/cv2624sup1.cif

Structure factors: contains datablocks I. DOI: 10.1107/S1600536809042056/cv2624Isup2.hkl

Additional supplementary materials:  crystallographic information; 3D view; checkCIF report

## Figures and Tables

**Table 1:** Hydrogen-bond geometry (Å, °)

*D*—H⋯*A*	*D*—H	H⋯*A*	*D*⋯*A*	*D*—H⋯*A*
N2—H4⋯O3^i^	0.88	2.06	2.912 (6)	161
N4—H3⋯O2^ii^	0.88	2.02	2.879 (6)	166

Symmetry codes: (i) 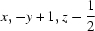; (ii) 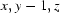.

**Fig. 1.**
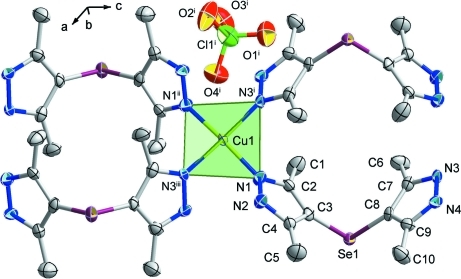
A portion of the crystal structure of the title compound showing the labeling scheme and 50% probabilty displacement ellipsoids [symmetry codes: (i) –x, 1–y, –z, (ii) –x, y, -0.5–z, (iii) x, 1–y, -1/2 + z]. H atoms are omitted for clarity.

**Fig. 2.**
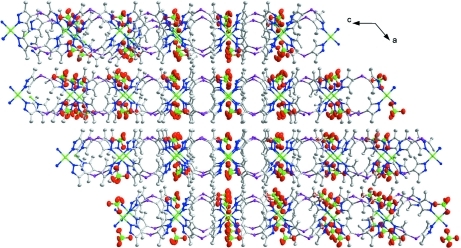
A packing diagram of the title compound viewed along the b-axis. H atoms are omitted for clarity.

## supplementary crystallographic information

### Comment

Molecular self-assembly through donor-acceptor interactions becomes one of the most elaborated research areas in coordination chemistry. The primary interest here is the development of functional materials with useful properties. Particularly, infinite molecular polymeric arrays are potentially applicable as specifically ordered crystalline substances with reversible selective sorption (Farha *et al.*, 2009), electrical conductivity (Shibahara *et al.*, 2007) and molecular magnetism functionality (Ohba *et al.*, 2009).

The title compound, [Cu(*cis*-µ-*L*)_2_](ClO_4_)_2_, was readily prepared by mixing aquoeous solution of Cu(ClO_4_)_2_.6H_2_O and methanolic solution of the ligand bis(3,5-dimethyl-1*H*-pyrazolyl)selenide (*L*) prepared according to Seredyuk *et al.* (2007). A tetragonally distorted square-planar environment of the Cu^II^ ion is formed by four non-coplanar nitrogen atoms of propeller-like arranged pyrazolyl cycles (distances Cu–N are 1.982 (5) and 1.967 (5) Å, two diagonal angles N–Cu–N are 163.6 (3) and 168.8 (3)°, respectively). Symmetrically equivalent ligand molecules in *cis*-bonding configuration are linked to Cu^II^ ion in a double-stranded bridge fashion (Fig. 1.). By repeats, they form linear chain running along the *c* axis within which each copper atom deviates from the average position by a value of ±0.068 (5) Å (Fig. 2). The NH group of each pyrazole cycle is involved in hydrogen bonding with perchlorate group resulting in the formation of a three-dimensional hybrid network.

### Experimental

A solution of Cu(ClO_4_)_2_.6H_2_O (0.065 g) in water (10 ml) was mixed with a solution of L.H_2_O (0.1 g) in methanol (10 ml) and was set aside for one week after which brown crystals of the title compound were isolated. Found C, 29.83, H, 3.65, N, 13.81. C_20_H_28_Cl_2_CuN_8_O_8_Se_2 _requires C, 29.99, H, 3.52, N, 13.99.

### Refinement

All H atoms were geometrically positioned (C—H  0.98 Å; N—H 0.88 Å), and refined as riding, with U_iso_(H) = 1.2-1.5 U_eq_(C, N). The crystal studied was a twin, so matrix (100) was used in the refinement of the crystal structure.

### Crystal data

**Table:**

[Cu(C_10_H_14_N_4_Se)_2_](ClO_4_)_2_	*F*(000) = 1596
*M**_r_* = 800.86	*D*_x_ = 1.758 Mg m^−^^3^
Monoclinic, *C*2/*c*	Mo *K*α radiation, λ = 0.71073 Å
Hall symbol: -C 2yc	Cell parameters from 3400 reflections
*a* = 28.398 (6) Å	θ = 2.9–27.5°
*b* = 7.5865 (15) Å	µ = 3.36 mm^−^^1^
*c* = 18.517 (4) Å	*T* = 120 K
β = 130.69 (3)°	Plates, brown
*V* = 3025.1 (17) Å^3^	0.2 × 0.15 × 0.05 mm
*Z* = 4	

### Data collection

**Table:**

Nonius KappaCCD diffractometer	3415 independent reflections
Radiation source: fine-focus sealed tube	2799 reflections with *I* > 2σ(*I*)
graphite	*R*_int_ = 0.074
ω–scans	θ_max_ = 27.5°, θ_min_ = 1.5°
Absorption correction: multi-scan (*SADABS*; Sheldrick, 1996)	*h* = −36→34
*T*_min_ = 0.552, *T*_max_ = 0.845	*k* = −9→9
13077 measured reflections	*l* = −24→22

### Refinement

**Table:**

Refinement on *F*^2^	Primary atom site location: structure-invariant direct methods
Least-squares matrix: full	Secondary atom site location: difference Fourier map
*R*[*F*^2^ > 2σ(*F*^2^)] = 0.047	Hydrogen site location: inferred from neighbouring sites
*wR*(*F*^2^) = 0.115	H-atom parameters constrained
*S* = 1.04	*w* = 1/[σ^2^(*F*_o_^2^) + (0.0574*P*)^2^ + 8.3063*P*] where *P* = (*F*_o_^2^ + 2*F*_c_^2^)/3
3415 reflections	(Δ/σ)_max_ < 0.001
191 parameters	Δρ_max_ = 2.18 e Å^−^^3^
0 restraints	Δρ_min_ = −1.00 e Å^−^^3^

### Special details

**Table:**

Geometry. All e.s.d.'s (except the e.s.d. in the dihedral angle between two l.s. planes) are estimated using the full covariance matrix. The cell e.s.d.'s are taken into account individually in the estimation of e.s.d.'s in distances, angles and torsion angles; correlations between e.s.d.'s in cell parameters are only used when they are defined by crystal symmetry. An approximate (isotropic) treatment of cell e.s.d.'s is used for estimating e.s.d.'s involving l.s. planes.
Refinement. Refinement of *F*^2^ against ALL reflections. The weighted *R*-factor *wR* and goodness of fit *S* are based on *F*^2^, conventional *R*-factors *R* are based on *F*, with *F* set to zero for negative *F*^2^. The threshold expression of *F*^2^ > σ(*F*^2^) is used only for calculating *R*-factors(gt) *etc*. and is not relevant to the choice of reflections for refinement. *R*-factors based on *F*^2^ are statistically about twice as large as those based on *F*, and *R*- factors based on ALL data will be even larger.

### Fractional atomic coordinates and isotropic or equivalent isotropic displacement parameters (Å^2^)

**Table:**

	*x*	*y*	*z*	*U*_iso_*/*U*_eq_	
Cu1	0.0000	0.48037 (13)	−0.2500	0.0124 (2)	
Se1	0.15222 (2)	0.73993 (6)	0.14892 (4)	0.01476 (14)	
Cl1	0.13436 (8)	0.99852 (19)	0.36166 (12)	0.0307 (4)	
O1	0.1340 (3)	1.0280 (6)	0.2844 (4)	0.0369 (12)	
O2	0.1537 (3)	1.1562 (6)	0.4173 (3)	0.0430 (13)	
O3	0.1753 (2)	0.8577 (5)	0.4177 (4)	0.0410 (14)	
O4	0.0732 (2)	0.9468 (7)	0.3248 (4)	0.0510 (14)	
N1	0.0655 (2)	0.5177 (6)	−0.1109 (3)	0.0144 (10)	
N2	0.1167 (2)	0.4166 (6)	−0.0546 (3)	0.0146 (10)	
H4	0.1257	0.3301	−0.0755	0.017*	
N3	0.0637 (2)	0.5450 (6)	0.2384 (3)	0.0125 (10)	
N4	0.1183 (2)	0.4511 (6)	0.2931 (3)	0.0131 (10)	
H3	0.1284	0.3739	0.3367	0.016*	
C1	0.0190 (3)	0.7707 (8)	−0.0923 (4)	0.0237 (14)	
H11A	0.0036	0.8098	−0.1548	0.036*	
H11B	0.0367	0.8713	−0.0486	0.036*	
H11C	−0.0155	0.7211	−0.0987	0.036*	
C2	0.0679 (3)	0.6330 (7)	−0.0538 (4)	0.0141 (12)	
C3	0.1216 (3)	0.6022 (7)	0.0402 (4)	0.0128 (12)	
C4	0.1526 (3)	0.4643 (7)	0.0377 (4)	0.0146 (11)	
C5	0.2128 (3)	0.3741 (9)	0.1141 (5)	0.0268 (15)	
H19A	0.2057	0.2792	0.1420	0.040*	
H19B	0.2422	0.4595	0.1637	0.040*	
H19C	0.2300	0.3245	0.0867	0.040*	
C6	0.0155 (3)	0.7705 (8)	0.1110 (5)	0.0217 (14)	
H8A	−0.0160	0.7091	0.0508	0.033*	
H8B	0.0328	0.8680	0.1000	0.033*	
H8C	−0.0035	0.8168	0.1362	0.033*	
C7	0.0657 (3)	0.6460 (7)	0.1804 (4)	0.0140 (12)	
C8	0.1218 (3)	0.6135 (7)	0.1993 (4)	0.0128 (11)	
C9	0.1539 (3)	0.4907 (7)	0.2723 (4)	0.0148 (12)	
C10	0.2162 (3)	0.4049 (8)	0.3247 (5)	0.0250 (14)	
H17A	0.2105	0.2792	0.3088	0.037*	
H17B	0.2407	0.4188	0.3935	0.037*	
H17C	0.2380	0.4608	0.3061	0.037*	

### Atomic displacement parameters (Å^2^)

**Table:**

	*U*^11^	*U*^22^	*U*^33^	*U*^12^	*U*^13^	*U*^23^
Cu1	0.0104 (4)	0.0185 (5)	0.0111 (5)	0.000	0.0082 (4)	0.000
Se1	0.0156 (2)	0.0179 (3)	0.0128 (3)	−0.0062 (2)	0.0101 (3)	−0.0039 (2)
Cl1	0.0342 (8)	0.0231 (7)	0.0390 (9)	0.0019 (7)	0.0258 (8)	0.0063 (7)
O1	0.052 (3)	0.032 (3)	0.038 (3)	−0.003 (2)	0.034 (3)	−0.001 (2)
O2	0.068 (4)	0.030 (2)	0.027 (3)	0.000 (3)	0.029 (3)	0.003 (2)
O3	0.055 (4)	0.022 (2)	0.064 (4)	0.013 (2)	0.048 (3)	0.016 (2)
O4	0.037 (3)	0.070 (4)	0.048 (3)	−0.017 (3)	0.029 (3)	−0.011 (3)
N1	0.015 (3)	0.016 (2)	0.012 (2)	0.002 (2)	0.008 (2)	−0.0016 (18)
N2	0.013 (3)	0.016 (2)	0.012 (2)	0.0048 (19)	0.007 (2)	−0.0009 (18)
N3	0.012 (2)	0.018 (2)	0.010 (2)	0.004 (2)	0.008 (2)	0.0002 (18)
N4	0.011 (2)	0.015 (2)	0.010 (2)	0.0013 (19)	0.005 (2)	0.0022 (17)
C1	0.024 (4)	0.021 (3)	0.017 (3)	0.009 (3)	0.010 (3)	0.001 (2)
C2	0.012 (3)	0.017 (3)	0.016 (3)	0.001 (2)	0.011 (3)	0.001 (2)
C3	0.015 (3)	0.014 (3)	0.011 (3)	−0.003 (2)	0.009 (2)	0.001 (2)
C4	0.015 (3)	0.017 (2)	0.014 (3)	0.001 (2)	0.011 (3)	0.001 (2)
C5	0.021 (4)	0.033 (4)	0.024 (3)	0.007 (3)	0.013 (3)	0.003 (3)
C6	0.015 (3)	0.026 (3)	0.022 (3)	0.004 (3)	0.010 (3)	0.011 (2)
C7	0.017 (3)	0.014 (3)	0.011 (3)	−0.001 (2)	0.009 (3)	−0.001 (2)
C8	0.014 (3)	0.015 (3)	0.010 (3)	−0.001 (2)	0.009 (2)	−0.003 (2)
C9	0.012 (3)	0.017 (3)	0.016 (3)	−0.001 (2)	0.009 (2)	−0.001 (2)
C10	0.020 (3)	0.031 (3)	0.029 (3)	0.006 (3)	0.018 (3)	0.008 (3)

### Geometric parameters (Å, °)

**Table:**

Cu1—N3^i^	1.967 (5)	C1—H11A	0.9800
Cu1—N3^ii^	1.967 (5)	C1—H11B	0.9800
Cu1—N1	1.982 (5)	C1—H11C	0.9800
Cu1—N1^iii^	1.982 (5)	C2—C3	1.396 (8)
Se1—C8	1.893 (5)	C3—C4	1.387 (8)
Se1—C3	1.902 (5)	C4—C5	1.497 (8)
Cl1—O3	1.413 (5)	C5—H19A	0.9800
Cl1—O2	1.434 (5)	C5—H19B	0.9800
Cl1—O1	1.442 (5)	C5—H19C	0.9800
Cl1—O4	1.447 (5)	C6—C7	1.478 (8)
N1—C2	1.339 (7)	C6—H8A	0.9800
N1—N2	1.347 (6)	C6—H8B	0.9800
N2—C4	1.349 (7)	C6—H8C	0.9800
N2—H4	0.8800	C7—C8	1.413 (8)
N3—C7	1.349 (7)	C8—C9	1.386 (8)
N3—N4	1.373 (6)	C9—C10	1.502 (8)
N3—Cu1^i^	1.967 (5)	C10—H17A	0.9800
N4—C9	1.330 (7)	C10—H17B	0.9800
N4—H3	0.8800	C10—H17C	0.9800
C1—C2	1.497 (8)		
			
N3^i^—Cu1—N3^ii^	168.8 (3)	C4—C3—C2	106.5 (5)
N3^i^—Cu1—N1	91.28 (16)	C4—C3—Se1	126.0 (4)
N3^ii^—Cu1—N1	90.32 (16)	C2—C3—Se1	127.0 (4)
N3^i^—Cu1—N1^iii^	90.32 (16)	N2—C4—C3	105.9 (5)
N3^ii^—Cu1—N1^iii^	91.28 (16)	N2—C4—C5	121.9 (5)
N1—Cu1—N1^iii^	163.6 (3)	C3—C4—C5	132.2 (5)
C8—Se1—C3	101.66 (19)	C4—C5—H19A	109.5
O3—Cl1—O2	110.9 (3)	C4—C5—H19B	109.5
O3—Cl1—O1	108.4 (3)	H19A—C5—H19B	109.5
O2—Cl1—O1	109.5 (3)	C4—C5—H19C	109.5
O3—Cl1—O4	107.6 (3)	H19A—C5—H19C	109.5
O2—Cl1—O4	110.2 (4)	H19B—C5—H19C	109.5
O1—Cl1—O4	110.2 (4)	C7—C6—H8A	109.5
C2—N1—N2	106.7 (5)	C7—C6—H8B	109.5
C2—N1—Cu1	129.9 (4)	H8A—C6—H8B	109.5
N2—N1—Cu1	123.3 (3)	C7—C6—H8C	109.5
N1—N2—C4	111.7 (4)	H8A—C6—H8C	109.5
N1—N2—H4	124.1	H8B—C6—H8C	109.5
C4—N2—H4	124.1	N3—C7—C8	109.2 (5)
C7—N3—N4	105.7 (4)	N3—C7—C6	122.5 (5)
C7—N3—Cu1^i^	131.4 (4)	C8—C7—C6	128.3 (5)
N4—N3—Cu1^i^	122.9 (3)	C9—C8—C7	106.1 (5)
C9—N4—N3	112.0 (4)	C9—C8—Se1	126.9 (4)
C9—N4—H3	124.0	C7—C8—Se1	126.3 (4)
N3—N4—H3	124.0	N4—C9—C8	107.1 (5)
C2—C1—H11A	109.5	N4—C9—C10	120.8 (5)
C2—C1—H11B	109.5	C8—C9—C10	132.0 (5)
H11A—C1—H11B	109.5	C9—C10—H17A	109.5
C2—C1—H11C	109.5	C9—C10—H17B	109.5
H11A—C1—H11C	109.5	H17A—C10—H17B	109.5
H11B—C1—H11C	109.5	C9—C10—H17C	109.5
N1—C2—C3	109.1 (5)	H17A—C10—H17C	109.5
N1—C2—C1	121.8 (5)	H17B—C10—H17C	109.5
C3—C2—C1	129.1 (5)		
			
N3^i^—Cu1—N1—C2	−53.2 (5)	N1—N2—C4—C5	−178.8 (5)
N3^ii^—Cu1—N1—C2	138.0 (5)	C2—C3—C4—N2	−1.3 (6)
N1^iii^—Cu1—N1—C2	42.3 (5)	Se1—C3—C4—N2	−174.1 (4)
N3^i^—Cu1—N1—N2	122.9 (4)	C2—C3—C4—C5	178.5 (6)
N3^ii^—Cu1—N1—N2	−45.9 (4)	Se1—C3—C4—C5	5.7 (9)
N1^iii^—Cu1—N1—N2	−141.6 (4)	N4—N3—C7—C8	0.3 (6)
C2—N1—N2—C4	−0.4 (6)	Cu1^i^—N3—C7—C8	−179.0 (4)
Cu1—N1—N2—C4	−177.3 (4)	N4—N3—C7—C6	−178.8 (5)
C7—N3—N4—C9	0.7 (6)	Cu1^i^—N3—C7—C6	2.0 (9)
Cu1^i^—N3—N4—C9	180.0 (4)	N3—C7—C8—C9	−1.0 (6)
N2—N1—C2—C3	−0.4 (6)	C6—C7—C8—C9	177.9 (6)
Cu1—N1—C2—C3	176.2 (4)	N3—C7—C8—Se1	−172.0 (4)
N2—N1—C2—C1	178.4 (5)	C6—C7—C8—Se1	7.0 (9)
Cu1—N1—C2—C1	−5.0 (8)	C3—Se1—C8—C9	97.2 (5)
N1—C2—C3—C4	1.1 (6)	C3—Se1—C8—C7	−93.7 (5)
C1—C2—C3—C4	−177.6 (6)	N3—N4—C9—C8	−1.3 (6)
N1—C2—C3—Se1	173.8 (4)	N3—N4—C9—C10	179.1 (5)
C1—C2—C3—Se1	−4.9 (9)	C7—C8—C9—N4	1.4 (6)
C8—Se1—C3—C4	−91.4 (5)	Se1—C8—C9—N4	172.3 (4)
C8—Se1—C3—C2	97.3 (5)	C7—C8—C9—C10	−179.1 (6)
N1—N2—C4—C3	1.1 (6)	Se1—C8—C9—C10	−8.3 (9)

Symmetry codes: (i) −*x*, −*y*+1, −*z*; (ii) *x*, −*y*+1, *z*−1/2; (iii) −*x*, *y*, −*z*−1/2.

### Hydrogen-bond geometry (Å, °)

**Table:**

*D*—H···*A*	*D*—H	H···*A*	*D*···*A*	*D*—H···*A*
N2—H4···O3^ii^	0.88	2.06	2.912 (6)	161
N4—H3···O2^iv^	0.88	2.02	2.879 (6)	166

Symmetry codes: (ii) *x*, −*y*+1, *z*−1/2; (iv) *x*, *y*−1, *z*.
